# Peripheral Chorioretinal Imaging Through a Front Prism on Optical Coherence Tomography Angiography

**DOI:** 10.1167/tvst.10.14.36

**Published:** 2021-12-30

**Authors:** Kentaro Kawai, Tomoaki Murakami, Saori Sakaguchi, Tatsuya Yamada, Shin Kadomoto, Akihito Uji, Akitaka Tsujikawa

**Affiliations:** 1 Department of Ophthalmology and Visual Sciences, Kyoto University Graduate School of Medicine, Kyoto, Japan

**Keywords:** optical coherence tomography angiography, peripheral retina, prism, widefield imaging

## Abstract

**Purpose:**

To evaluate the clinical feasibility of peripheral chorioretinal imaging through a front prism on swept-source optical coherence tomography angiography (SS-OCTA).

**Methods:**

We prospectively obtained en face OCTA images using SS-OCTA in 10 eyes of 10 healthy volunteers. For the peripheral chorioretinal imaging, the scanning laser passed and refracted through a 45°−90°−45° right-angle prism. We evaluated the qualitative and quantitative characteristics of chorioretinal vessels in the periphery.

**Results:**

Using peripheral chorioretinal imaging through a prism, the retinal vasculature was delineated to the equator on the OCTA images, and varices of the vortex vein ampullae were observed on choroidal OCT images. The 3 × 3-mm images revealed three-dimensional morphologies unique to the peripheral vasculature, such as the gap between retinal arterioles and venules in the superficial capillary plexus (SCP) and elliptical and greater lobules in the choriocapillaris layer. Compared with OCTA images obtained without the prism, those obtained through the prism demonstrated an approximately 1.24-fold increase in the lengths in the base apex direction, whereas the lengths in the perpendicular direction showed concordance. The peripheral vessel density (VD) in the inferior quadrant was lower than those in the other quadrants on the SCP and deep capillary plexus, whereas on the SCP images of the macula the lowest VD was observed in the temporal subfield.

**Conclusions:**

Peripheral chorioretinal imaging allowed us to generate ultra-widefield panoramic OCTA images and demonstrated morphologic characteristics unique to peripheral chorioretinal vessels.

**Translational Relevance:**

OCTA imaging through a front prism can be a technique for acquiring chorioretinal vasculature images in the periphery.

## Introduction

Imaging of the retinal vasculature is generally feasible to delineate both ocular and systemic diseases.^1^ During retinal vasculature development, vascular endothelial cells originate from the optic disc and migrate toward the periphery, which is mediated via vascular endothelial growth factor (VEGF) expression, as retinal neuroglial cells develop.^2^^,^^3^ Histological studies have shown that the major trunks of arterioles and venules run mainly in the ganglion cell layer with bifurcation to the radial peripapillary capillary network in the nerve fiber layer and deep capillary plexuses in the innermost and outermost of the inner nuclear layer.^4^^–^^6^ Retinal vessels are asymmetric in the macula, nasal versus temporal subfields, and superficial versus deep layers, which may influence the pathogenic processes in glaucoma and retinal diseases.^7^^,^^8^ However, the in vivo characteristics of retinal vessels in each quadrant of the peripheral retina remain to be elucidated, in both healthy subjects and those with ocular diseases.

Color fundus photography is a standard method used to assess the morphology and color tone of major retinal vessels. Fluorescein angiography is a gold-standard imaging modality to evaluate the morphologic and functional characteristics mainly in superficial vessels, including capillaries.^9^^–^^11^ These modalities complementarily help us to assess pathogenic vascular changes. An ultra-widefield (UWF) imaging device has recently enabled imaging of almost the entire retina (up to 200°) in a single shot.^12^^–^^14^ It allows visualization of pathology, such as a nonperfusion area or retinal neovascularization, in the peripheral retina.

The clinical application of optical coherence tomography angiography (OCTA) has enabled us to evaluate the chorioretinal vasculature three dimensionally.^15^^,^^16^ OCTA offers a higher contrast; thus, it can provide readily quantifiable data.^11^ This noninvasive imaging has expanded our knowledge about pathological processes in the posterior pole in diseases of the chorioretinal vessels and optic disc. With the advancement in OCTA devices, such as a higher scan speed on swept-source (SS) OCTA and the widefield montage technique, the field of view is increasing and reaching to the mid-periphery.^8^ However, most of the commercially available OCTA machines do not allow us to assess vessels in the periphery, although several vascular diseases are characterized by lesions throughout the retina. In this study, we devised a method of acquiring OCTA images of the peripheral retina by refracting the OCT beam with a prism and preliminarily characterized the peripheral retinal vessels in healthy eyes using these images.

## Methods

### Participants

Ten eyes of 10 healthy volunteers were prospectively recruited into this study. The inclusion criteria were being a healthy adult (≥20 years old) and providing informed consent. The exclusion criteria were a history of ocular or systemic diseases, poor quality of SS-OCTA images in the macula (signal strength index of 7 or less), and axial length of < 22mm or >26 mm. All study procedures adhered to the tenets of the Declaration of Helsinki and were performed with the approval of the Kyoto University Graduate School and Faculty of Medicine Ethics Committee. Written informed consent was obtained from all participants after a full explanation of the nature of the study.

### OCTA Image Acquisition

After an interview about the history of diseases and a comprehensive ophthalmic examination, we quantified the axial length using partial coherence interferometry (IOLMaster; Carl Zeiss Meditec, Jena, Germany). Fundus images were acquired under pupil dilatation with 0.5% tropicamide and 0.5% phenylephrine hydrochloride. We obtained UWF color fundus photographs using an Optos 200Tx (Optos PLC, Dunfermline, UK). OCTA imaging was performed using the PLEX Elite 9000 (Carl Zeiss Meditec). PLEX Elite 9000 operates at 100,000 A-scans per second with a swept laser source (central wavelength of 1040 to 1060 nm). Both nominal 6 × 6-mm and 12 × 12-mm images were made of 500 × 500 A-scans and exported at a size of 1024 × 1024 pixels.^8^ Nominal 3 × 3-mm images were constructed from 300 × 300 A-scans and converted to 1024 × 1024-pixel digital resolution.

**Figure 1.**
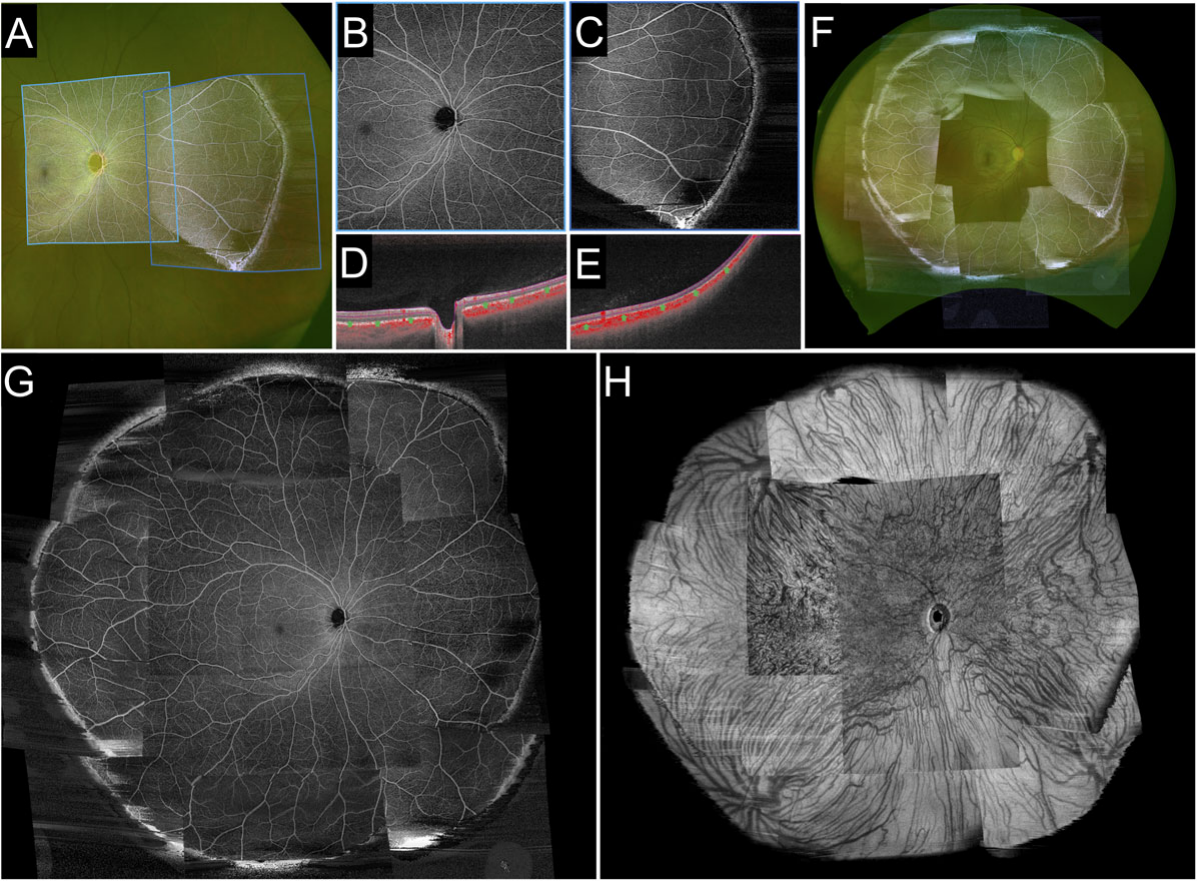
Peripheral chorioretinal imaging through a 45°−90°−45° right-angle prism. (A) En face OCTA images acquired using an internal target at 15° (*left square*; in the device, the internal target could only be shifted by 15°) and in the periphery obtained through the prism (*right square*) were fitted onto the corresponding areas in the UWF photograph. (B, C) Raw images of the SCP and (D, E) the corresponding B-scan images with flow signal (*red*) along the *green arrows*. (F) Montage of eight peripheral OCTA images of the SCP acquired with a 12 × 12-mm scan pattern. The prism base was oriented in eight directions (every 45°). (G) Montage images of the posterior pole and the peripheral images in eight directions allowed us to create UWF-OCTA images to the equator. (H) Corresponding structural OCT image of the choroid slab. Varices of the vortex vein ampullae were also delineated.

To obtain OCTA images in the peripheral regions, the OCT beam was refracted with a front prism. Participants wore an optometry trial frame equipped with a 45°−90°−45° right-angle prism (Sigmakoki Co., Ltd., Tokyo, Japan) and a fixation light (external tiny light emitting diode) at approximately 15°. The optical glass prism was made of BK7 glass and bent the OCT beam coming perpendicularly from the device by about 26°. Participants were instructed to fixate the external light with the eye to be examined. The OCT beam was aimed to the center of the prism to achieve peripheral chorioretinal imaging (Figs. 1A–1E). The prism base was oriented in four directions (nasal, superficial, temporal, and inferior quadrants) for the 6 × 6-mm images or in one direction (temporal quadrant) for the 12 × 12-mm images.

We generated four en face OCTA images: (1) the superficial capillary plexus (SCP), from the internal limiting membrane to the inner plexiform layer (IPL); (2) the deep capillary plexus (DCP), from the IPL to 110 µm above the Bruch's membrane; (3) the choriocapillaris layer, from 6 to 26 µm posterior to the retinal pigment epithelium; and (4) the choroid layer, from 64 to 115 µm posterior to Bruch's membrane). The default setting of the manufacturer's software was used, except for the choriocapillaris slab. The IPL was defined as the layer at 70% of the inner thickness between the internal limiting membrane and the outer plexiform layer. Manual corrections were performed only for the images used for the montage OCTA images shown in Figure 1. Projection artifacts from superficial vessels were removed using the manufacturer's software before exporting the DCP images.

In order to show how the field of view changes by peripheral chorioretinal imaging with a 12 × 12-mm scan pattern, we created montage OCTA images based on the UWF color fundus photographs using ImageJ (National Institutes of Health, Bethesda, MD), as recently described (Figs. 1A, 1F–1H).^17^ Briefly, corresponding vascular bifurcations were manually chosen on both 12 × 12-mm OCTA images and UWF color fundus photographs, and non-linear transformation of OCTA images was performed using the Landmark Correspondences ImageJ plug-in. The deformed OCTA images were pasted onto the UWF photographs to generate the panoramic OCTA images using the Z Project function. By applying the peripheral chorioretinal imaging technique and using the 45°−90°−45° right-angle prism, we could observe the retinal vessels to the equator on the OCTA images of the SCP (Fig. 1F). In the choroidal slabs of the structural OCT images in the corresponding areas, varices of the vortex vein ampullae were also delineated. The montage images of the posterior pole and the peripheral images obtained in eight directions enabled us to create UWF-OCTA images to the equator (Figs. 1F–1H).

### Image Distortion Evaluation

**Figure 2.**
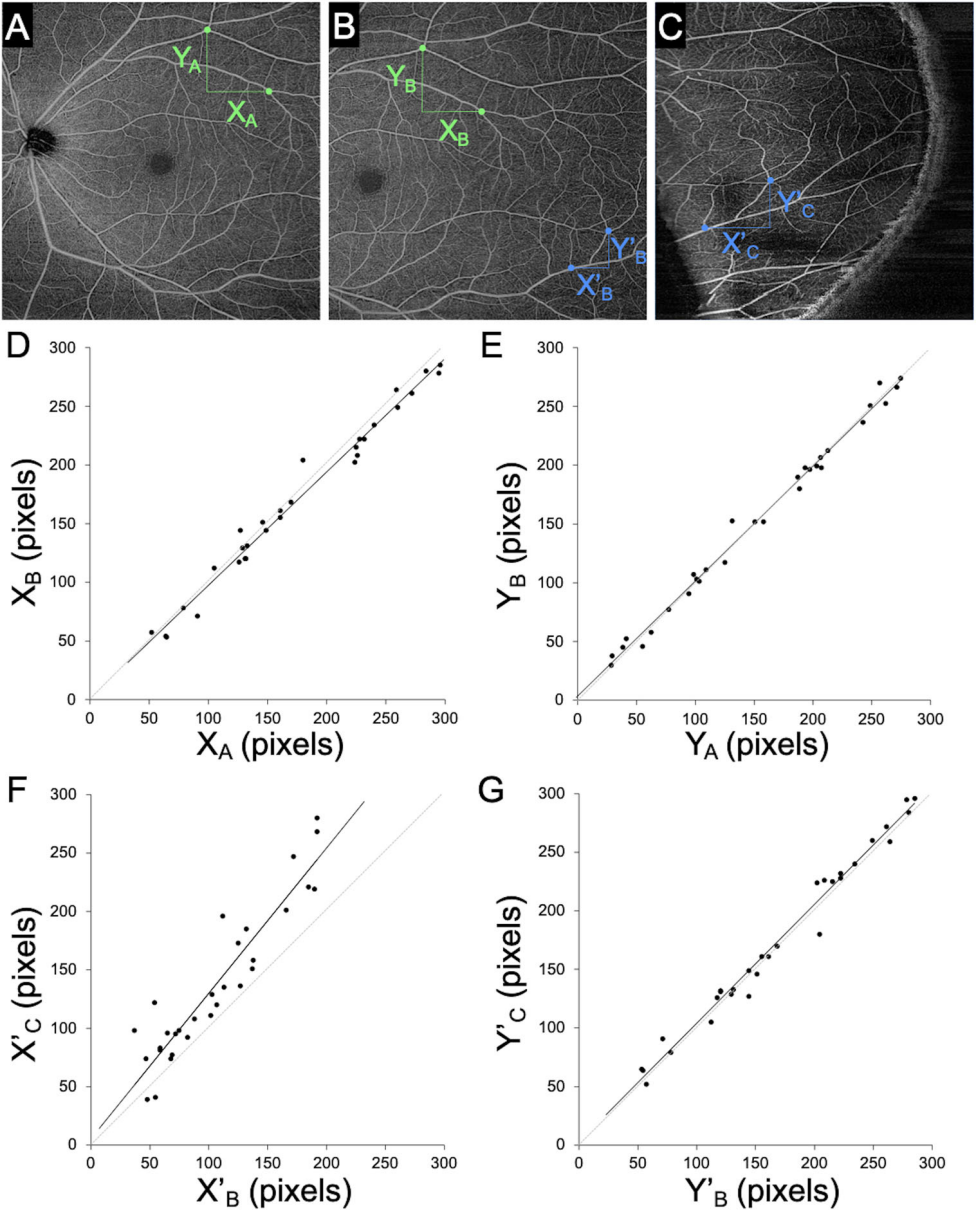
Image distortion in the base apex direction in images obtained using the peripheral chorioretinal imaging technique. (A) The 12 × 12-mm scan pattern OCTA image centered on the fovea. (B) The 12 × 12-mm OCTA image fixated to the internal target at 15°. (C) The 12 × 12-mm OCTA image in the periphery obtained by peripheral chorioretinal imaging. *X* = distance between two vascular branching points in the base apex direction. *Y* = distance between two vascular branching points in the perpendicular direction. (D) Concordance of the base apex distances between panels A and B. (E) Concordance of the perpendicular distances between panels A and B. (F) Concordance of the base apex distances between panels B and C; *X*′*_C_* = 1.24 × *X*′*_B_* + 5.49. (F) Concordance of the perpendicular distances between panels B and C.

**Figure 3.**
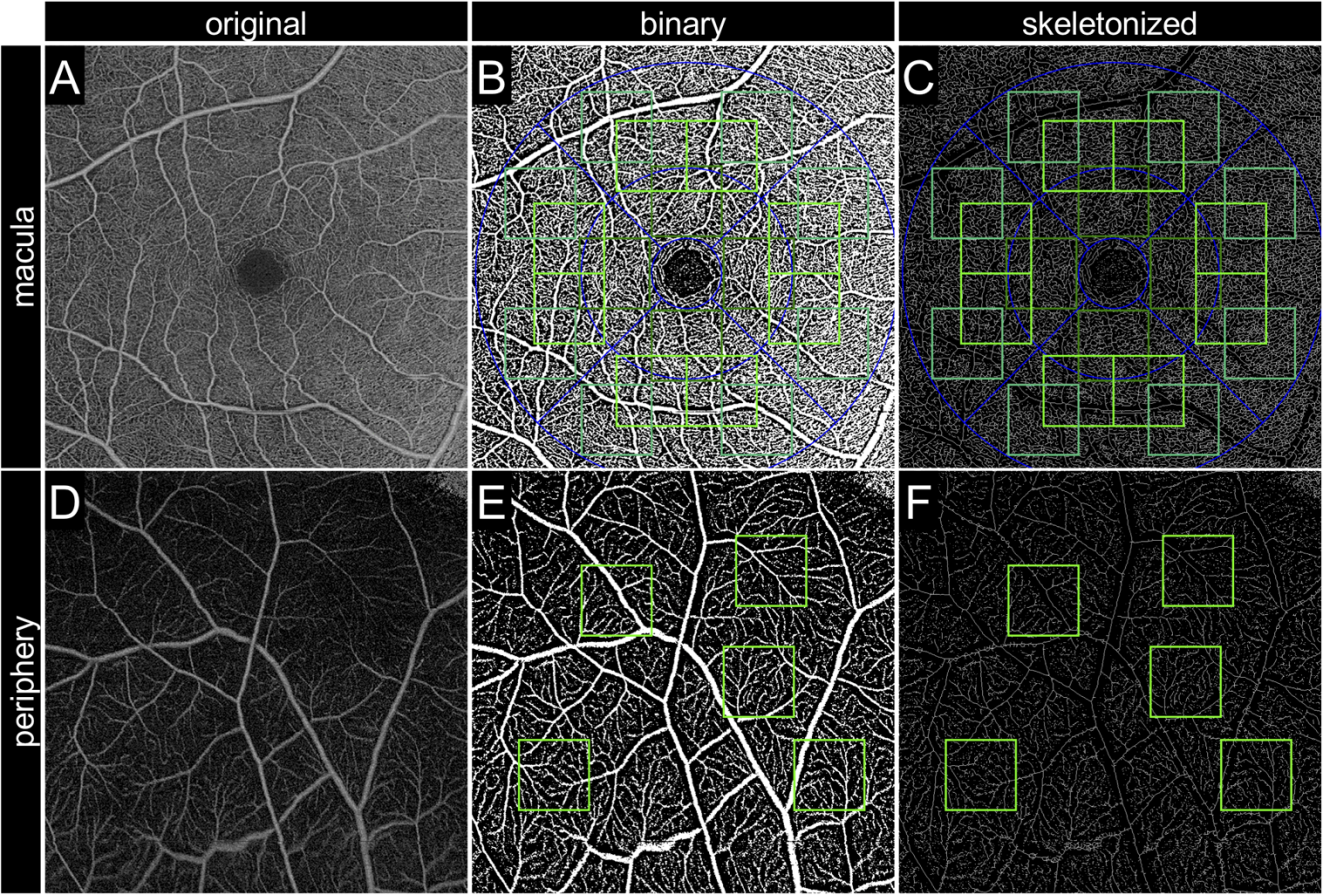
Image processing for the quantification of vascular parameters. (A, D) En face OCTA images obtained with a 6 × 6-mm scan pattern in the SCP and the corresponding binary (B, E) and skeletonized (C, F) images. A to C are 6 × 6-mm squares centered on the fovea. D to F are 6 × 6-mm peripheral images obtained by peripheral chorioretinal imaging. The *green squares* represent ROIs for quantification. The *blue line* represents the Early Treatment Diabetic Retinopathy Study grid (1-, 3-, and 6-mm diameters).

We acquired three 12 × 12-mm OCTA images: (1) centered on the fovea (Fig. 2A), (2) in the temporal quadrant fixated to the internal target at 15° (Fig. 2B), and (3) in the temporal quadrant using the peripheral chorioretinal imaging technique through the prism (Fig. 2C). In the SCP images, two vessel bifurcation points were selected in the areas shared by two images after the exclusion of motion artifacts and segmentation error. The horizontal (*X_A_* vs. *X_B_* or *X*′*_B_* vs. *X*′*_C_*) and vertical (*Y_A_* vs. *Y_B_* or *Y*′*_B_* vs. *Y*′*_C_*) distances that corresponded to the lengths in the base apex and its perpendicular directions, respectively, were compared in two images to analyze the distortion in images obtained under fixation to the internal target or by peripheral chorioretinal imaging through the prism. For each image, three pairs of vessel bifurcations were selected, followed by measurement of the pixel distances using ImageJ. The corresponding distances between different images were compared.

### Quantification of Vascular Parameters

We quantified three vascular parameters in four quadrants (nasal, superior, temporal, and inferior) on OCTA images acquired with a 6 × 6-mm scan pattern centered on the fovea and those obtained using the peripheral chorioretinal imaging technique in the SCP and DCP. In the OCTA images of the macula, five nominal 1 × 1-mm squares (170 × 170 pixels) were determined as the regions of interest (ROIs) in each quadrant, as shown in the upper row in Figure 3. The centers of the inner, middle, or outer squares were 175, 297, and 437 pixels from the fovea, respectively. In the peripheral retina, five squares with 170 × 170 pixels were determined as ROIs at random in areas without segmentation errors or motion artifacts. The average values of the five ROIs were used for comparison between regions. The distances from the fovea to ROIs in the peripheral retina were measured using a built-in Optos 200Tx tool. These distances were not significantly different between quadrants (12.4 ± 2.2 mm in the nasal quadrant, 12.8 ± 3.7 mm in the upper quadrant, 12.0 ± 1.6 mm in the temporal quadrant, and 11.9 ± 1.4 mm in the lower quadrant; *P* = 0.448).

For quantitative measurement of the vessel density (VD), exported images were processed in three steps: level correction, binarization, and pixel counting. After image import into ImageJ, the minimum displayed value was altered from 0 to 50 or 40 in the SCP or DCP images, respectively, in order to reduce the background signals. The binarized images were generated using the Phansalkar adaptive local thresholding method (white and black pixels representing the blood vessels and background, respectively).^18^ The white pixels were counted using the Analyze Particles function and divided by the total pixels to define the ratio of the areas occupied by vessels as the VD in this study.^19^^–^^21^

The vessel length density (VLD) was calculated as the ratio of the area occupied by skeletonized vessels to the total area. The aforementioned binary images were applied to the Skeletonize function, followed by the counting of white pixels. The fractal dimension (FD), which represents the complexity of the vasculature, was calculated on the skeletonized images using the Fractal Box Count function.^19^^–^^21^

### Statistical Analysis

All values were expressed as the mean ± standard deviation. The Shapiro–Wilk test was applied to confirm normal distribution. The concordance and correlation were analyzed using intraclass correlation coefficients (ICCs) and Pearson's correlation coefficient, respectively. Differences were assessed using analysis of variance with Bonferroni's correction. *P* < 0.05 was considered statistically significant. All analyses were performed using SPSS Statistics 24 (IBM Corporation, Armonk, NY).

## Results

The mean age of the participants was 31.3±10.4 years; five participants were men. The peripheral chorioretinal imaging technique using the front prism allowed us to obtain the slab images of retinal vessels and choroidal structure to the equator (Fig. 1).

### Image Distortion

We evaluated the image distortion in the peripheral images obtained through the prism. The comparative studies revealed that, despite the best concordance in the perpendicular distances (ICC = 0.993; 95% confidence interval [CI], 0.986–0.997) (Fig. 2E), the distances in the base apex direction differed between the images obtained with and those obtained without the prism (ICC = 0.892; 95% CI, 0.787–0.947) (Fig. 2D). There was a significant association (*R* = 0.930; *P* < 0.001), and the regression line was *X*′*_C_* = 1.24 × *X*′*_B_* + 5.49. In contrast, the comparison between images centered on the fovea and those in the temporal quadrant fixated to the internal target at 15° (Figs. 2A, 2B) demonstrated concordance of the lengths in the base apex and perpendicular directions (ICC = 0.993; 95% CI, 0.986–0.997 vs. ICC = 0.996; 95% CI, 0.992–0.998, respectively). Distortion was evaluated in the DCP images, as well, and the results were almost the same as in the SCP images (Supplementary Fig. S1). These results suggest image extension in the base apex direction in the peripheral images obtained through the prism.

### Vascular Characteristics of the Peripheral Retina

**Figure 4.**
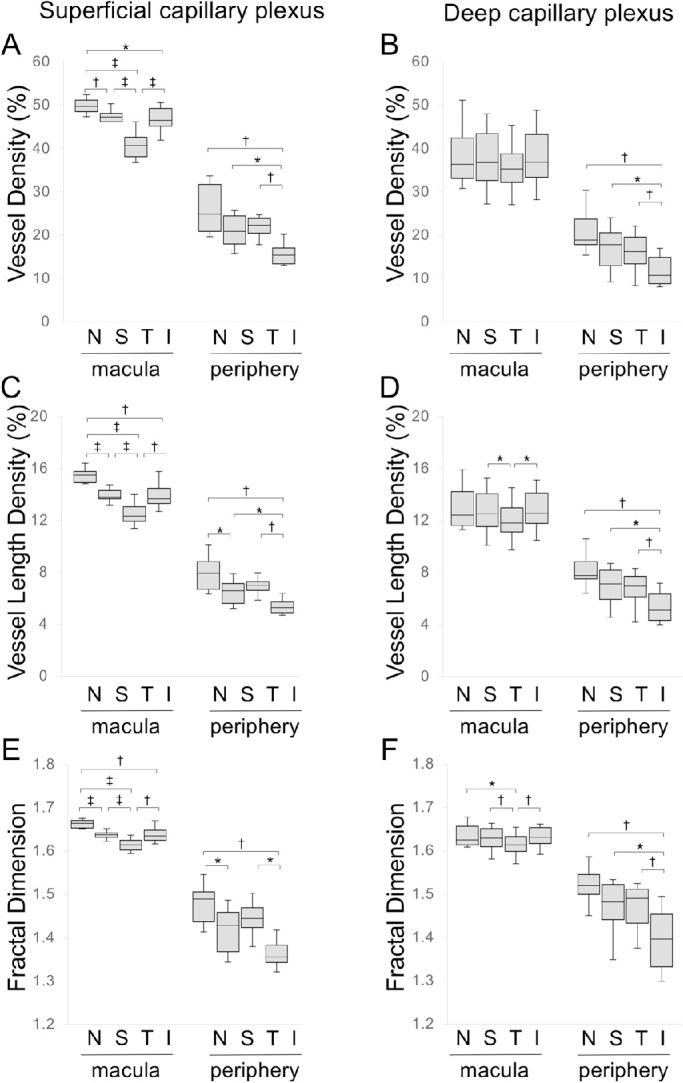
Vascular parameters in each quadrant of the macula and peripheral region. (A, B) Vessel density. (C, D) Vessel length density. (E, F) Fractal dimension in each quadrant in the SCP (A, C, E) and DCP (B, D, F). N, nasal; T, temporal; S, superior; I, inferior. The macula is a 6 × 6-mm square centered on the fovea. The periphery is a 6 × 6-mm scan pattern image obtained by peripheral chorioretinal imaging. ^*^P < 0.05. ^†^*P* < 0.01. ^‡^*P* < 0.001.

We investigated the qualitative and quantitative characteristics in the retinal vasculature on the SCP and DCP images obtained by the peripheral chorioretinal imaging technique. The peripheral retina had lower VD, VLD, and FD in the SCP and DCP than those in the macula in each quadrant (Fig. 4). On the SCP images of the peripheral retina, these parameters were lower in the inferior than in the nasal and temporal quadrants (Figs. 4A, 4C, 4E). In contrast, the VDs in the nasal and temporal quadrants were the highest and lowest in the macula, respectively. On the DCP images of the peripheral regions, the VD, VLD, and FD in the inferior quadrant were lower than those in other quadrants, whereas there were no differences in the VD among the four quadrants of the macula (Figs. 4B, 4D, 4F).

**Figure 5.**
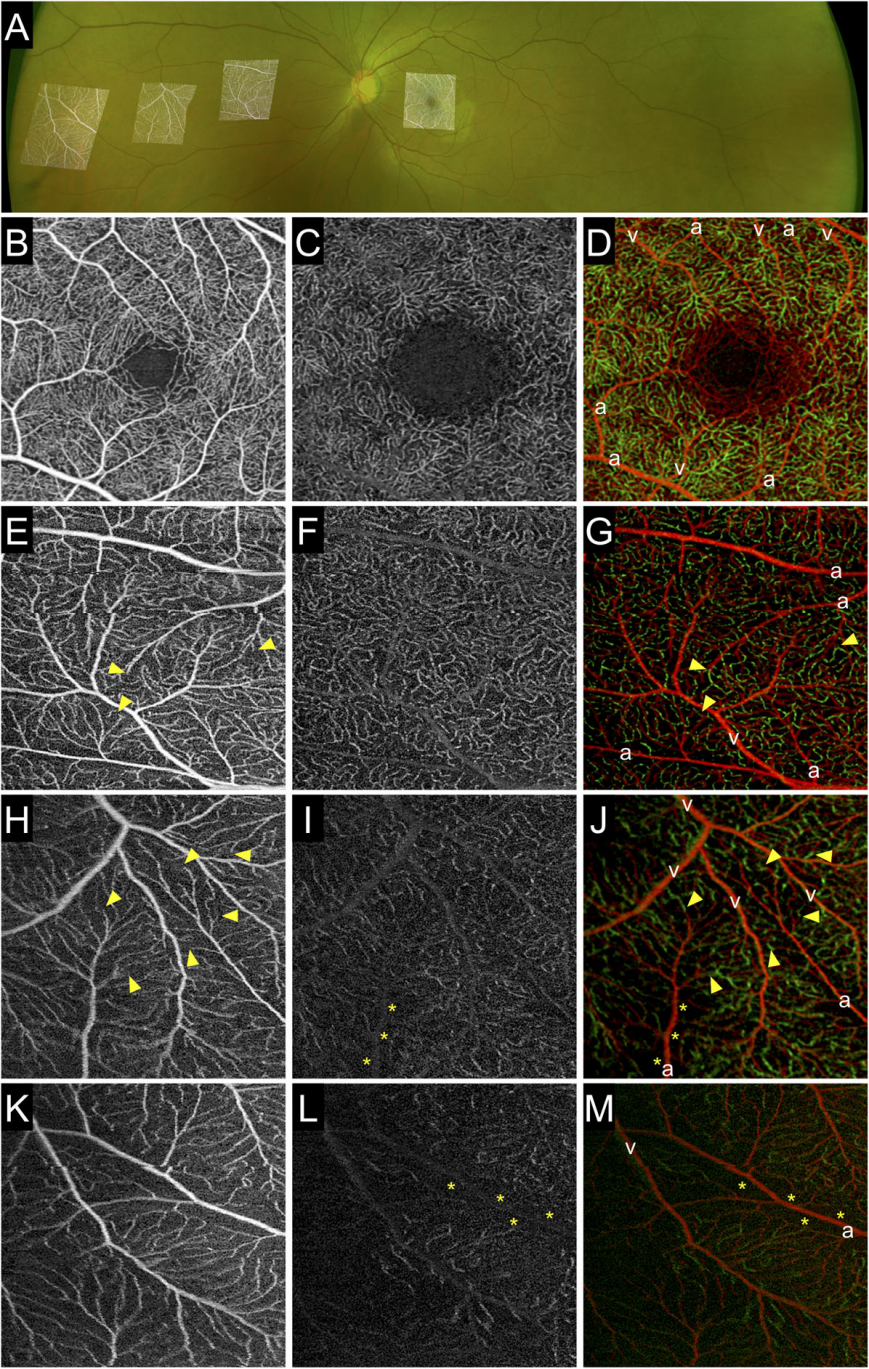
Qualitative characteristics of the retinal vessels in the far periphery. (A) The 3 × 3-mm scan pattern OCTA images obtained by peripheral chorioretinal imaging were fitted onto the UWF fundus photograph. (B-D) En face images in the macula. (E-M) Enface images in the extramacular regions—mid-periphery (E–G), equator (H–J), and far periphery (K–M)—obtained using a 45°−90°−45° right-angle prism and individual fixations. (B, E, H, K) En face OCTA images in the SCP. (C, F, I, L) En face OCTA images in the DCP. The gaps between arterioles and venules in the SCPs were connected via capillaries in the deep layer (*arrowheads*). Capillary-free zones were delineated beneath arterioles in the DCP (*asterisks*). Capillaries appeared to be straight in the periphery, compared with the meshwork in the macula. (D, G, J, M) The Gaussian blur function in ImageJ (radius = 2) was applied to images of the SCP and DCP, followed by the creation of merged images. *Red*, superficial capillaries; *green*, deep capillaries; a, arteriole/artery; v, venule/vein.

**Figure 6.**
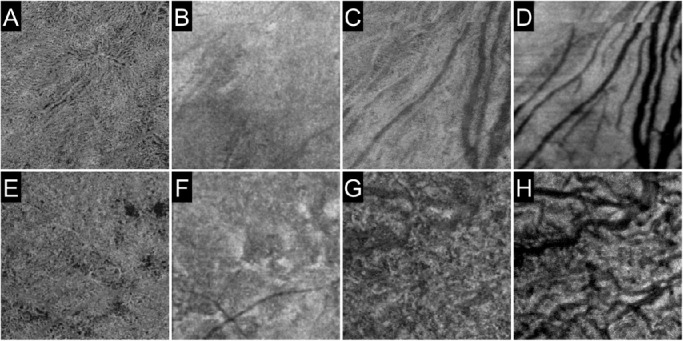
Choriocapillaris and choroid in the periphery. The 3 × 3-mm scan pattern en face images in the choriocapillaris (A, B, E, F) and choroid (C, D, G, H) layers were obtained by peripheral chorioretinal imaging. The Gaussian blur function in ImageJ (radius = 2) was applied to all images. Vessels in the choriocapillaris slab are delineated more clearly in the inferior quadrant (A–D) than in the temporal quadrant (E–H). Images A, C, E, and G are OCTA images, whereas images B, D, F, and H are the corresponding structural OCT images.

Qualitative assessment in the 3 × 3-mm retinal slabs revealed that there were sometimes gaps between arterioles and venules in the SCP, which were connected via capillaries in the DCP (Figs. 5E–5J), compared with the artery-capillary-vein unit in the SCP in the macula (Fig. 5B). The capillaries in the DCP were absent beneath the arterioles of the peripheral region, as shown in the horizontal capillary-free zone around arterioles in the SCP (Figs. 5H–5M). Superficial capillaries appeared to be straighter in the periphery than those in the macula (Figs. 5H, 5K). In the 3 × 3-mm choriocapillaris slab, the vascular meshwork was often connected to feeding vessels, whereas there was no apparent vascular structure in the macula (Figs. 6A, 6E).

## Discussion

Several ophthalmologic diseases, such as retinitis pigmentosa and diabetic retinopathy, are characterized by morphological changes in the periphery first, although commercially available OCTA machines cannot delineate the peripheral microcirculation.^22^^,^^23^ In order to delineate peripheral retinas, two approaches may make the OCT beam bend to the periphery: shift of visual fixation and refraction of the OCT beam per se. The range of eye movement is limited, and an involuntary saccade often occurs at the extreme gaze shifts.^24^ We therefore proposed a new technique of OCTA through a prism—peripheral chorioretinal imaging—which enabled us to evaluate vessels quantitatively and to visualize the microcirculation in living human peripheral retina (Supplementary Fig. S2). Intriguingly, the VD was reduced in the periphery, particularly in the inferior quadrant in healthy subjects. OCTA images in the periphery delineated gaps between arterioles and venules in the SCP and absence of deep capillaries beneath arterioles. Future studies should be planned to elucidate whether these quantitative and qualitative characteristics influence the development or progression of retinal vascular diseases.

A previous study showed that the superficial VD in a 4.5-mm circle at a distance of 5 mm from the fovea was higher than that in the macular region.^25^ This finding may be explained by the presence of a thick nerve fiber layer along the vascular arcades containing radial peripapillary capillaries. In contrast, the current study examined the more peripheral retina (12.3 ± 2.4 mm from the fovea) and demonstrated that the VD, VLD, and FD were significantly lower in the periphery than those in the macula, as indicated by a decrease in the inner retinal thickness to the periphery, although it should be noted that the quantification of peripheral images was somewhat affected by distortion due to the prism. The coordinated expression of VEGF is necessary for the development of superficial vessels to the periphery and the accompanying deep capillaries.^3^^,^^26^ We speculate that the lower VD or VLD may be a result of lower levels of VEGF expression and reduced perfusion pressure in the thinner retina periphery during vascular development. The VD was lower in the inferior quadrant than in other quadrants of the peripheral region. In contrast, the VD was greater in the nasal subfield than in the temporal subfield of the macula. This suggests that vascular development is modulated by different mechanisms in the macula and periphery. It remains to be investigated whether these findings in the inferior quadrant of the periphery are related to an optic fissure (choroid fissure) during organogenesis, inferior posterior staphyloma, or posterior coloboma in the same quadrant.^27^

This study documented qualitative findings in the peripheral region: a capillary-free zone beneath arterioles, straight capillaries, and a gap between arterioles and venules in the SCP. Straight capillaries and the gap between arterioles and venules may share a common mechanism, vascular regression.^28^ Generally, primary vascular plexuses are pruned depending on blood flow and several angiogenic molecules.^29^ As retinal ganglion cells, the main source of VEGF, are reduced during development, a deficiency of VEGF might lead to local regression of capillaries and subsequent vascular gaps.^30^^,^^31^ In addition, the greater blood flow in straight capillaries might contribute to the selection for their own survival. Arteriogenesis and synchronized vascular pruning around the arteries sculpt the periarterial capillary-free zone in the retina,^28^^,^^32^ which forms horizontally in the SCP, whereas deep capillaries appear to be a seamless network in the thick macula. In contrast, capillary-free zones beneath arterioles were present vertically in the DCP of the thin peripheral region. This suggests that diffusible factors rather than intrinsic molecules contribute to development of the capillary-free zone, although the underlying molecular mechanisms remain ill-defined.^28^

These qualitative and quantitative characteristics may influence the development or progression of retinal vascular diseases. Lower VD, straight appearance of capillaries, and accompanying deep vascular layers may regulate the blood flow and perfusion pressure and modify the development of capillary nonperfusion.^33^ The gap between arterioles and venules in the SCP might not allow the lamellar obstruction of blood flow, whereas lamellar non-perfused areas were observed in the macula. Peripheral arterioles may also correspond to perfusion boundaries, such as those in the mid-periphery.^34^ Peripheral chorioretinal imaging often, but not always, delineated the in vivo vascular meshwork and its feeding vessels on the choriocapillaris OCTA slab. Greater capillaries and intercapillary spaces allow delineation of the choriocapillaris in the periphery, compared with no apparent vessels in the macula. It is consistent with the histological angioarchitecture; in the periphery, precapillary arterioles and postcapillary venules were accompanied with capillaries in the plane of the choriocapillaris.^35^^,^^36^ Future studies should elucidate how the diversity of vascular morphologies contributes to pathogenesis in chorioretinal vascular diseases.

Despite the great advantage of peripheral imaging, the image distortion is a concern. The imaging through the 45°−90°−45° right-angle prism led to image extension in the base apex direction but not in its perpendicular direction. This extension ratio might vary depending on the position within the images, the relative position of the eye to the device, or asymmetric optic media (e.g., cornea, crystalline lens, curvature of the posterior segments). By this distortion, vessels running in the base apex direction or its perpendicular direction might appear to be longer or to have a greater diameter, respectively, thus suggesting that this imaging technique interferes with quantification of the VLD and FD. The parameters in this study might be relative but not absolute values, and future studies should investigate the methodologies necessary to adjust the image distortion.

Our study has further limitations. First, this preliminary study with a small number of cases just showed the clinical feasibility of peripheral chorioretinal imaging through a prism. In the future, prospective large-scale studies should confirm the normative data and elucidate the pathological changes in the peripheral vasculature. We got the impression that automatic segmentation by the equipped software was often inaccurate in the periphery. The real-time eye-tracking system in this OCTA machine did not function through the prism, which resulted in frequent motion artifacts. Second, residual superficial projection artifacts may still have some effect on DCP imaging despite the use of projection removal software, as noted in a previous study.^37^ These factors would reduce the quality of image assessments.^38^^,^^39^ Third, the prism might cause dispersion. Full bandwidth of the PLEX Elite 9000 is 1000 to 1100 nm, and the refractive indexes of the prism used in this study for light at 1000 nm and 1100 nm were 1.508 and 1.507, respectively. Therefore, the difference in refracting angle by wavelength may be minimal. Furthermore, vascular parameters in this study cannot be compared directly with those obtained in previous studies in which other image processing methods were employed. To date, there is no consensus on methods to quantify vascular parameters on OCTA images.^40^

In this study, we proposed a novel imaging technique, peripheral chorioretinal imaging through a prism, to acquire OCTA images of the peripheral chorioretinal vessels. The preliminary data documented that the peripheral retina has different density, location, and morphologies of vessels compared with the macula in healthy subjects.

## Supplementary Material

Supplement 1

Supplement 2
