# Supplementary material for: Peripheral Chorioretinal Imaging Through a Front Prism on Optical Coherence Tomography Angiography
Source: Transl Vis Sci Technol. 2021 Dec 30;10(14):36. doi: 10.1167/tvst.10.14.36 (PMC8727309; doi:10.1167/tvst.10.14.36)
Supplement: Supplement 1 [file tvst-10-14-36_s001.pdf]

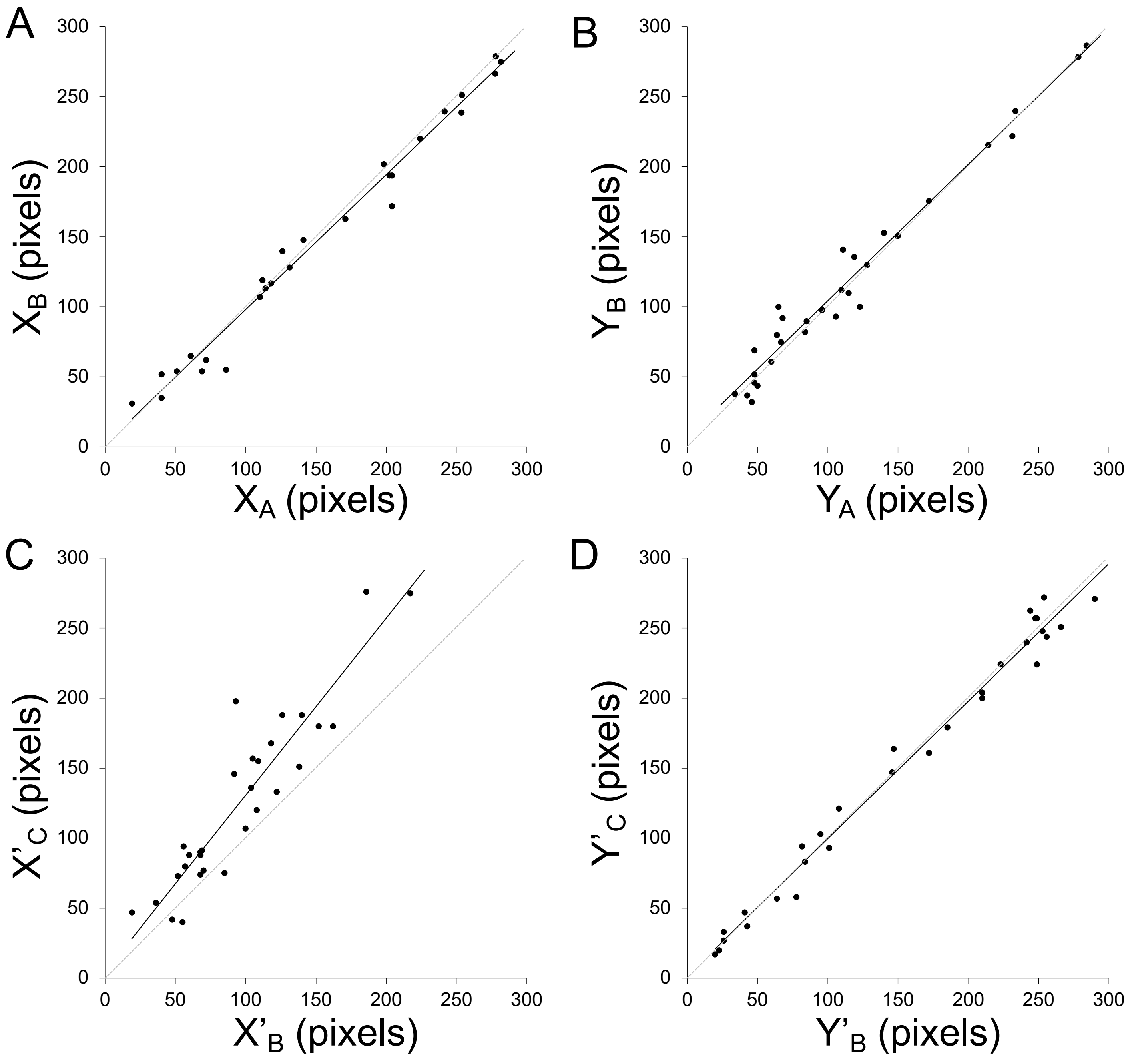

Supplementary Figure 1. Image distortion in deep capillary plexus in images obtained using the peripheral chorioretinal imaging technique.

(A-D) Concordance of the base-apex and perpendicular distances in deep capillary plexus corresponding to D-G in Figure 2, respectively. (A) Concordance of the base-apex distances between optical coherence tomography angiography (OCTA) images centered on the fovea and OCTA images fixated to the internal target at 15° (ICC = 0.991, 95% CI, 0.980-0.996). (B) Concordance of the perpendicular distances between OCTA images centered on the fovea and OCTA images fixated to the internal target at 15° (ICC = 0.984, 95% CI, 0.967-0.992). (C) Concordance of the base-apex distances between OCTA images fixated to the internal target at 15° and OCTA images in the periphery obtained by peripheral chorioretinal imaging (R=0.922, P<0.001). $X'_C = 1.26 \times X'_B + 4.14$. (D) Concordance of the perpendicular distances between OCTA images fixated to the internal target at 15° and OCTA images in the periphery obtained by peripheral chorioretinal imaging (ICC = 0.992, 95% CI, 0.984-0.996).
