# Supplementary material for: Peripheral Chorioretinal Imaging Through a Front Prism on Optical Coherence Tomography Angiography
Source: Transl Vis Sci Technol. 2021 Dec 30;10(14):36. doi: 10.1167/tvst.10.14.36 (PMC8727309; doi:10.1167/tvst.10.14.36)
Supplement: Supplement 2 [file tvst-10-14-36_s002.pdf]

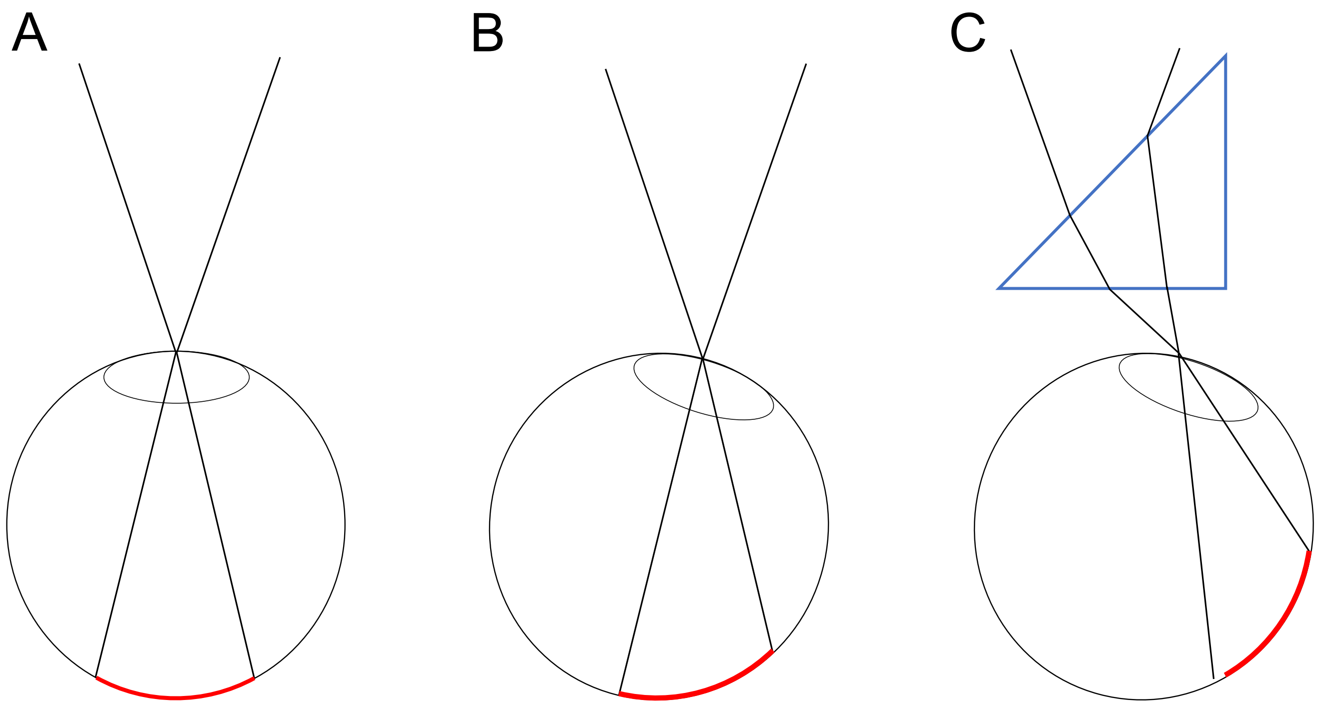

Supplementary Figure 2. The scheme of peripheral chorioretinal imaging.
The red line shows the range in which the OCTA image can be acquired. (A) Acquisition of the OCTA image centered on the fovea. (B) The OCTA image is steered to the periphery by shifting the fixation target. (C) The OCTA image is further steered to the periphery by bending the OCT beam with a prism in addition to shifting the fixation light. Blue triangle; prism.
